# Impacts of Three Gorges Reservoir on the sedimentation regimes in the downstream-linked two largest Chinese freshwater lakes

**DOI:** 10.1038/srep35396

**Published:** 2016-10-17

**Authors:** Yongqiang Zhou, Erik Jeppesen, Jingbao Li, Yunlin Zhang, Xinping Zhang, Xichun Li

**Affiliations:** 1 Taihu Laboratory for Lake Ecosystem Research, State Key Laboratory of Lake Science and Environment, Nanjing Institute of Geography and Limnology, Chinese Academy of Sciences, Nanjing 210008, China; 2 University of Chinese Academy of Sciences, Beijing 100049, China; 3 Sino-Danish Centre for Education and Research, Beijing 100190, China; 4 Department of Bioscience and Arctic Research Centre, Aarhus University, DK-8600 Silkeborg, Denmark; 5 College of Resources and Environmental Sciences, Hunan Normal University, Changsha 410081, China; 6 Hunan Hydro & Power Design Institute, Changsha 410007, China

## Abstract

We studied the impacts of Three Gorges Reservoir (TGR) on the sedimentation regimes in the downstream-linked two largest Chinese freshwater lakes, Lake Dongting and Lake Poyang. Our results indicate that up to 1.73 × 10^9^ t sediment was retained in TGR from June 2003 to December 2014. This resulted in a 145.9 × 10^6^ t yr^−1^ decline in the suspended sediment load at Zhicheng and a 16.8 × 10^6^ t yr^−1^ lower sediment flow from Yangtze River to Lake Dongting, which partially explains the 13.4 × 10^6^ t yr^−1^ lower sedimentation in Lake Dongting during the post-TGR period. Furthermore, TGR resulted in a 0.5 ± 0.3 m reduction of the multi-year mean water level at the Lake Poyang outlet Hukou, accelerating the suspended sediment export discharge from the lake. The reduced sedimentation in Lake Poyang during the post-TGR period was estimated to 6.3 × 10^6^ t yr^−1^. We estimate that a monthly mean concentration of sediment flow from TGR below 0.60 kg m^−3^ will lead to erosion in Lake Dongting and Lake Poyang. Better regulation of TGR may extend the life expectancy of the two vanishing large lakes.

For decades worldwide, enhanced anthropogenic use of land has resulted in increased terrestrial sediment erosion with subsequently higher loading to and accumulation of sediment in downstream-linked riverways and deltas^1^,^2^,^3^. However, river damming, diversion projects and recently improved land cover practices have had a counteracting effect, creating reduced accumulation and even, in some places, erosion of downstream river beds or deltas^2^,^4^,^5^,^6^,^7^,^8^,^9^. In recent decades, especially the deposition of suspended sediment in rivers has become increasingly affected by dam constructions^10^,^11^,^12^,^13^,^14^,^15^,^16^. In consequence, more focus has been directed at changes in suspended sediment load regimes in river channels and estuaries of upstream damming. Human control of aquatic resources via daming has profound human impacts on aquatic ecosystems^5^,^8^,^17^,^18^,^19^,^20^,^21^. Due to damming, a large quantity of suspended sediment is retained in the upstream reservoirs. In this way, less suspended sediment is discharged from the reservoir to the downstream-linked lakes, which become gradually eroded^1^,^2^,^19^. The effect of Three Gorges Reservoir (TGR) on net sedimentation/erosion in the downstream-linked Lake Dongting and Lake Poyang is today scarcely documented, but our study will add useful knowledge to that which is currently available^4^,^22^.

The two largest freshwater lakes in China, Lake Dongting and Lake Poyang located in the middle reaches of Yangtze River, provide drinking water to a population of at least 10 million in the surrounding areas^23^. Lake Dongting receives water and suspended sediment from the Yangtze River via three channels (located immediately downstream of Zhicheng), four rivers (Xiang, Zi, Yuan and Li) and ungauged small tributaries, and the lake discharges water and suspended sediment to Yangtze River at Chenglingji (Fig. 1). The suspended sediment input from the three channels constitutes the majority of suspended sediment sources to Lake Dongting^19^,^24^,^25^. In comparison, Lake Poyang receives water and suspended sediment from five rivers (Gan, Fu, Xin, Rao and Xiu) and ungauged small rivers, and, after the regulation and storage, it discharges suspended sediment into the Yangtze River at Hukou (Fig. 1). Vast amounts of sediment have been accumulated in the two lakes during the past century^4^,^19^,^22^,^26^. In combination, sedimentation, land reclamation and water abstraction in the surrounding areas have led to a major reduction of the lake volume^19^,^26^,^27^, from 293 × 10^8^ m^3^ in 1949 to 167 × 10^8^ m^3^ in 1995 (a 43% decrease) for Lake Dongting, and from 370 × 10^8^ m^3^ in 1953 to 261 × 10^8^ m^3^ in 1985 (a 29.5% decrease) for Lake Poyang (Fig. S1). Several sub-basins of the two lakes, for instance Lake Qili and Lake Muping in the western part of Lake Dongting, have even disappeared during the last three decades^22^,^27^. In consequence, the flood storage capacities of the two lakes have declined^27^,^28^,^29^, which partially explains the severe flood disaster in which 3704 people lost their lives in 1998 in the surrounding areas of the two lakes^30^,^31^,^32^.

In November 1997, the Yangtze River was closed to allow commencement of the construction of TGR, the world’s largest hydro-power facility, which came into operation in June 2003. Suspended sediment loads from upstream watersheds have largely been deposited within the reservoir itself, meaning that less suspended sediment is discharged from the reservoir in its outflow. This has led to erosion of downstream-linked river channels^4^,^12^,^21^,^25^,^33^,^34^,^35^,^36^,^37^. Whether TGR may extend the life expectancy of the two lakes remains unknown. Furthermore, few studies have been carried out to investigate the impact of the role of the operation of the once (pre-TGR) largest dam in China, Gezhouba (GZB), on the suspended sediment budgets of downstream-linked river reaches^4^,^19^,^38^, and this is therefore a subject of discussion in this study. Considering the large area of the ungauged regions, the contribution of ungauged suspended sediment to the sediment budget of the Yangtze River is an important factor to consider. This contribution has been quantified^19^,^26^ and estimated in some studies^25^,^33^, but the ungauged sediment source and its impact on the river-lake system has often not been element of investigation in previous studies^22^,^34^,^39^, resulting in an underestimation of the sediment deposition in downstream river reaches and lakes. This means that an accurate estimation of the suspended sediment budgets of Lake Poyang and Lake Dongting impacted by the operation of TGR is not available. Given that sedimentation in the two lakes may be highly influenced by the operation of TGR^4^,^22^,^25^, it is also important to identify the best way of regulating TGR to maintain an erosion-dominated state in the two lakes. So far, how to best regulate dams to maintain an erosion state in downstream-linked lakes has been a subject of limited investigation^4^,^22^.

In this study, we evaluated to which extent the establishment of TGR has simultaneously affected the sediment budgets of Lake Dongting and Lake Poyang by comparing the observed sediment budgets in the two lakes with a calculated non-TGR scenario (post-TGR). The threshold concentration of outflow suspended sediment from TGR to maintain an erosion state in the two lakes was estimated based on the relationships between the monthly mean concentration of suspended sediment in the TGR outflow at the Huanglingmiao outlet and the corresponding monthly sediment deposition/erosion rate in the two lakes. We hypothesise that the operation of TGR has contributed importantly to the observed trend of decreasing net deposition of sediment in the two lakes.

## Results

### Sediment retained by TGR

The amount of sediment deposited in TGR varied with the inflow suspended sediment loads to TGR. From June 2003 to December 2014, 1.73 × 10^9^ t sediment was retained (taking the load from ungauged areas into account, Fig. 2), and the mean deposition proportion (the ratio of deposition to sediment load) increased rapidly from 60.8 ± 2.3% in 2003–2005 to 84.6 ± 5.9% in 2006–2014 (Fig. 2). These results are similar to those of Yang *et al*.^4^,^19^. The outflow suspended sediment load from GZB was similar to the load from TGR, confirming that a close relationship exists between these two (*p* < 0.001, Fig. S2).

### Comparison between observed sediment budgets in the pre- and post-TGR periods

The annual mean suspended sediment load of Yangtze River at Zhicheng decreased significantly from the pre-TGR period (1951–2002, 500.8 ± 124.5 × 10^6^ t yr^−1^) to the post-TGR period (2003–2014, 49.5 ± 41.6 × 10^6^ t yr^−1^, a 90.1% decrease, *t*-test, *p* < 0.001, Fig. 3, Table S1). The annual suspended sediment load from Yangtze River to Lake Dongting via the three channels decreased markedly from the pre-TGR period (134.7 ± 68.2 × 10^6^ t yr^−1^, 77.9% of the total load to the lake) to the post-TGR period (10.3 ± 7.0 × 10^6^ t yr^−1^, 50.0% of the total load, a 92.4% decrease, *t*-test, *p* < 0.001, Fig. 3, Table S1). A close relationship was found between the annual suspended sediment import from Yangtze River to Lake Dongting via the three channels and the corresponding annual sediment deposition in the lake (1951–2014, *D*_Dongting_ = 0.98*S*_Three channels_ −3.41, *r*^2^ = 0.97, *p* < 0.001, Fig. S2). The annual suspended sediment load from the four rivers (Xiang, Zi, Yuan, Li) and the ungauged small rivers decreased pronouncedly from 38.3 ± 18.1 × 10^6^ t yr^−1^ in the pre-TGR period to 10.3 ± 5.2 × 10^6^ t yr^−1^ in the post-TGR period (a 73.1% decrease, *t*-test, *p* < 0.001). The annual suspended sediment output discharge from the Chenglingji outlet decreased notably from the pre-TGR period (43.0 ± 17.3 × 10^6^ t yr^−1^) to the post-TGR period (18.9 ± 5.6 × 10^6^ t yr^−1^, a 56.0% decrease, *t*-test, *p* < 0.001, Fig. 3, Table S1). This resulted in a significant decrease in the multi-year mean annual sediment deposition in Lake Dongting from the pre-TGR period (130.1 ± 66.6 × 10^6^ t yr^−1^, 75.2% of the total load) to the post-TGR period (1.7 ± 12.1 × 10^6^ t yr^−1^, 8.3% of the total load, a 98.7% decrease, *t*-test, *p* < 0.001, Fig. 3, Table S1).

For Lake Poyang, the annual suspended sediment load from the tributaries to the lake decreased significantly from 18.6 ± 7.4 × 10^6^ t yr^−1^ in the pre-TGR period (1956–2002) to 7.4 ± 4.0 × 10^6^ t yr^−1^ in the post-TGR period (2003–2014, a 60.2% decrease, *t*-test, *p* < 0.001, Fig. 3, Table S1). However, the mean annual outflow suspended sediment discharge at Hukou, the outlet of Lake Poyang, increased notably but not significantly from the pre-TGR period (9.7 ± 4.9 × 10^6^ t yr^−1^) to the post-TGR period (12.3 ± 3.7 × 10^6^ t yr^−1^, a 26.8% increase, *t*-test, *p* = 0.06, Fig. 3, Table S1). Accordingly, the annual sediment deposition in Lake Poyang decreased significantly from the pre-TGR period (9.4 ± 5.9 × 10^6^ t yr^−1^, 51.0% of the total load) to the post-TGR period (−4.9 ± 3.6 × 10^6^ t yr^−1^ (“−” represents erosion), −66.0% of the total load, a 152.1% decrease, *t*-test, *p* < 0.001, Fig. 3, Table S1). The observed multi-year mean water level of the Hukou outlet of Lake Poyang decreased considerably from 13.0 ± 0.9 m in the pre-TGR period (1950–2002) to 12.2 ± 0.9 m in the post-TGR period (2003–2014, *t*-test, *p* = 0.013; Fig. S3).

### Comparison between the observed and the non-TGR scenario (post-TGR)

The differences between the observed sediment budgets in the two lakes during the post-TGR period and the non-TGR scenario (post-TGR) were the impacts of TGR operation.

The observed multi-year mean suspended sediment load of the Yangtze River at Zhicheng and the sediment loads from the main river to Lake Dongting via the three channels were significantly lower in the post-TGR period (49.5 ± 41.6 × 10^6^ t yr^−1^ and 10.3 ± 7.0 × 10^6^ t yr^−1^, respectively) than in the non-TGR scenario (195.4 ± 71.3 × 10^6^ t yr^−1^ and 27.1 ± 13.0 × 10^6^ t yr^−1^, respectively, *t*-test, *p* < 0.005, Fig. 3; Table S1). Our results therefore imply that up to 145.9 × 10^6^ t yr^−1^ and 16.8 × 10^6^ t yr^−1^ of the decline in the suspended sediment load at Zhicheng and from the main river to Lake Dongting via the three channels, respectively, were induced by the operation of TGR. The observed multi-year mean suspended sediment outflow discharge from the Chenglingji outlet to Lake Dongting in the post-TGR period (18.9 ± 5.6 × 10^6^ t yr^−1^) was notably but not significantly lower than in the non-TGR scenario (22.3 ± 0.5 × 10^6^ t yr^−1^, *t*-test, *p* = 0.06, Fig. 3; Table S1). Accordingly, the observed multi-year mean sediment deposition in Lake Dongting in the post-TGR period (1.7 ± 12.1 × 10^6^ t yr^−1^, 8.3% of the total load) was significantly lower than in the non-TGR scenario (15.1 ± 14.8 × 10^6^ t yr^−1^, 73.5% of the total load; *t*-test, *p* < 0.001, Fig. 3; Table S1). The reduced mean annual sediment deposition rate in Lake Dongting induced by TGR operation during the post-TGR period was estimated to 13.4 × 10^6^ t yr^−1^ (Fig. 3; Table S1).

For Lake Poyang, however, the observed multi-year mean suspended sediment outflow discharge from the Hukou outlet in the post-TGR period (12.3 ± 3.7 × 10^6^ t yr^−1^) was significantly higher than in the non-TGR scenario (6.0 ± 1.2 × 10^6^ t yr^−1^, *t*-test, *p* < 0.001, Fig. 3; Table S1). Accordingly, the observed multi-year mean sediment deposition in Lake Poyang in the post-TGR period (−4.9 ± 3.6 × 10^6^ t yr^−1^, −66.0% of the total load) was significantly lower than that in the non-TGR scenario (1.4 ± 2.8 × 10^6^ t yr^−1^, 18.7% of the total load, *t*-test, *p* < 0.001, Fig. 3; Table S1). The decreased mean annual sediment deposition rate in Lake Poyang induced by TGR operation during the post-TGR period was therefore estimated to 6.3 × 10^6^ t yr^−1^ (Fig. 3; Table S1). The observed multi-year mean water level of the Hukou outlet to Lake Poyang in the post-TGR period (12.2 ± 0.9 m) was notably but not significantly lower than in the non-TGR scenario in the post-TGR period (12.7 ± 0.8 m, *t*-test, *p* = 0.18, Fig. S3).

### Seasonal sediment budgets of the two lakes in the post-TGR period

The observed monthly net sediment deposition period in Lake Dongting decreased from May-September 2003–2005 to June-August 2006–2014 and the multi-year mean sedimentation in the monthly net deposition period declined significantly from 23.3 ± 7.9 × 10^6^ t in 2003–2005 to 6.8 ± 5.3 × 10^6^ t in 2003–2005 (*t*-test, *p* < 0.001, Fig. S4). Also, there was net erosion throughout 2011. In the non-TGR scenario, the monthly net sediment deposition period decreased from May-September 2003–2005 to June-September 2006–2014 (Fig. S4). The reduced sedimentation in Lake Dongting induced by TGR operation mainly concentrated in the flood period (June-August, Fig. S4).

In Lake Poyang, the monthly net sediment deposition concentrated in May-July, i.e. earlier than in Lake Dongting, and both absolute deposition and the extent of erosion were much lower than in Lake Dongting (Fig. S4).

Significant relationships were recorded between the monthly suspended sediment load of the main river at the TGR outlet Huanglingmiao and the monthly sediment deposition rate in the downstream-linked Lake Dongting and Lake Poyang (*p* < 0.001, Fig. 4). Similarly, significant correlations were recorded between the suspended sediment concentration at the Huanglingmiao outlet and the monthly sediment deposition rate in the two lakes (*p* < 0.001, Fig. 4).

## Discussion

A substantial amount of suspended sediment has been retained by TGR after its operation and has intensified since 2003, reflecting the increased water storage capacity of the dam. This increase has been especially pronounced since 2008 when the water level of TGR rose to >172 m (Fig. 2). Moreover, the operation of TGR has contributed importantly to the decreased sediment deposition and enhanced erosion in Lake Dongting and Lake Poyang. This is evidenced (Fig. 3; Table S1) by 1) a significantly lower mean annual sediment deposition in Lake Dongting and Lake Poyang in the post-TGR period than in the non-TGR scenario, 2) the sudden diminished sedimentation in both lakes from the pre- to the post-TGR period, 3) the significantly lower multi-year mean suspended sediment load of Yangtze River at Zhicheng and the reduced suspended sediment load from the main river to Lake Dongting via the three channels in the post-TGR than in the pre-TGR period and, finally, 4) the significantly higher multi-year mean suspended sediment output discharge from the Lake Poyang outlet Hukou observed during the post-TGR period than in the non-TGR scenario. Further evidence comes from the close relationships observed between the monthly suspended sediment export discharge from TGR at Huanglingmiao and the sediment deposition rate in Lake Dongting and Lake Poyang (Fig. 4), as earlier demonstrated for Lake Dongting^4^,^22^,^25^,^40^. Several studies have shown that construction of dams results in decreased sedimentation in downstream-linked riverways and river deltas^1^,^6^,^7^,^16^,^19^,^20^,^25^,^39^,^41^,^42^,^43^,^44^, and our study illustrates that large dams will also lead to decreased sedimentation and enhanced erosion in downstream-linked lakes. The operation of TGR has had a much wider importance than GZB for the decrease in sediment deposition in the two lakes. This is reflected in the negligible suspended sediment load retained by GZB relative to TGR after the latter came into operation (Fig. 2) as well as the fact that the slope of the linear fitting between the inflow and outflow suspended sediment discharge from GZB was approximately 1 (Fig. S2).

The approach used in our study to estimate the sediment budgets in the non-TGR scenario for the two lakes is more comprehensive than those used in earlier studies in this region. By including the estimated input from the ungauged tributaries (assuming similar sediment yield rates as for the neighbouring gauged stations)^12^,^19^,^25^,^26^, we obtained a more accurate sediment budget for the lakes than with traditional approaches^22^,^34^,^39^. Furthermore, the monthly data from January 1992 to May 2003 reflect the most recent time period after the operation of GZB and before the operation of TGR, and their much higher temporal resolution allowed us to unravel the monthly sedimentation regimes in Lake Dongting (Fig. S4). However, the empirical relationship between *SI*_Poyang_ and *D*_Poyang_ used for Lake Poyang had a lower determination coefficient (*r*^2^ = 0.74) than that for Lake Dongting (*r*^2^ > 0.98), implying that the budgets are somewhat uncertain. Another uncertainty is that newly built dams (Fig. S5)^1^,^45^, decreased rainfall (Fig. S6) and improved land use practices, resulting in elevated forest coverage in the upstream Yangtze River^46^,^47^,^48^,^49^ after 2003, may in combination have enhanced the erosion in the middle reach of Yangtze River, potentially creating changes in the sedimentation regimes in the two lakes in the post-TGR period. Nevertheless, the empirical relationships (Equations (9), (11), (14) and (16) based on observed data in the pre-TGR period) that we used to estimate the sediment budgets in Lake Dongting and Lake Poyang for the non-TGR scenario in the post-TGR period were well validated using the observed data in the post-TGR period (Fig. S7). This implies that the changes in the hydraulic connection of the river-lake system induced by the newly built dams (except for TGR), decreased rainfall and improved land use practices in the upper reach of Yangtze River were of minor importance. We also assumed that the suspended sediment yield rates from the ungauged small watersheds were similar to those in the neighbouring gauged regions, possibly giving a slight underestimation of the sedimentation in the two lakes^19^,^26^ as no decreasing trend in the suspended sediment yield rates from the ungauged small catchments has been recorded during the last six decades^4^. The underestimation is, however, considered to be of minor importance due to the low proportion of ungauged areas in both lake basins as well as the low sediment yield rates of the ungauged areas.

The mechanisms responsible for the effect of TGR on the sediment budgets of Lake Dongting and Lake Poyang differ substantially. The TGR operation resulted in a substantial decrease in the suspended sediment load from the main river to Lake Dongting via the three channels (Fig. 3), the primary source of the total inflow suspended sediment flux to Lake Dongting during the past half century^19^,^22^. TGR operation caused erosion (Fig. S8) in the downstream-linked riverway^1^,^12^,^18^,^19^,^22^,^25^,^26^,^33^,^38^,^41^,^45^,^50^ and thereby lowered the suspended sediment load from the main river to Lake Dongting via the three channels^19^,^22^,^25^,^26^. Therefore, the suspended sediment deposition rate of Lake Dongting decreased notably^19^,^25^. In Lake Poyang, however, the TGR operation produced a notable decrease in the water level at the Hukou outlet (Fig. S9), especially during the inundation period^28^,^29^,^51^,^52^, which accelerated the suspended sediment export discharge from the lake to Yangtze River (Fig. 3). This led to a decreasing occurrence of storms event-induced transport of suspended sediment into the lake from the main river during the flood season^29^,^51^,^52^,^53^,^54^. Intensified sand dredging in Lake Poyang upstream of Hukou during the post-TGR period further stimulated the suspended sediment export discharge from the lake^34^,^55^, this being especially pronounced in 2003–2007^55^. This was substantiated by the observed significantly higher multi-year mean suspended sediment export discharge from Hukou during the post-TGR period than in the non-TGR scenario (Fig. 3, Table S1). The markedly higher multi-year mean suspended sediment export discharge from Hukou during the post-TGR period than in the pre-TGR period (Fig. 3; Table S1) provides further evidence of this.

Enhanced sediment erosion is expected to persist in Lake Dongting and Lake Poyang for the foreseeable future. Due to the decreasing rainfall in the area upstream of the Yangtze River Basin and the two lake basins (Fig. S6). An increasing number of river damming and diversion projects and improved land cover practices in the upstream watersheds will also contribute to enhance the erosion in downstream-linked waters^1^,^4^,^35^,^46^,^47^,^48^. In addition, large-scale illegal (and also legal) sand dredging in the two lakes may further augment the erosion in the two lakes^28^,^55^. However, the substantial sediment volume retained in TGR implies that its role in decreasing sedimentation and enhancing erosion in the downstream-linked Lake Dongting and Lake Poyang will diminish in the foreseeable future.

Based on the relationships calculated between the observed monthly mean concentration of suspended sediment in the TGR outflow at the Huanglingmiao outlet and the monthly sedimentation in Lake Dongting and Lake Poyang, the threshold for the monthly mean suspended sediment concentration export from TGR to maintain a state of erosion in both Lake Dongting and Lake Poyang was estimated to <0.60 kg m^−3^ (Fig. 3). The threshold is based on the assumption that no changes have occurred in the hydrological connection between the mainstream of Yangtze River and the two lakes during the post-TGR period. This threshold is much easier to use in practical dam operation than sediment load^22^. Better regulation of TGR may help enhance erosion and thus extend the life expectancy of the two large lakes that are at high risk of vanishing due to sedimentation and land reclamation^1^,^19^,^44^.

## Methods

### Data sources

Data on the daily water level of TGR, inflow rates to the TGR and GZB reservoirs as well as the corresponding outflow rates since 1 June 2003 were obtained from the hydrological information service system of the Three Gorges Corporation (http://www.ctg.com.cn/inc/sqsk.php#1643). Information on the long-term (1951–2014) daily flow rate and monthly runoff and data on suspended sediment load for the gauging stations in the Yangtze River Basin were obtained from the Hunan Hydro & Power Design Institute, the Hunan hydrological data service system (http://www.hnsw.com.cn/tabid/230/Default.aspx), the Hubei hydrological data service system (http://219.140.162.169:8800/rw4/report/fa02.asp) and the Yangtze River Water Conservancy Committee (YRWCC; http://www.cjw.gov.cn/zwzc/bmgb/). The measurements of flow rate and suspended sediment delivery rate are presented in detail in Yang *et al*.^4^, giving a 10–30 vertical array representation of profiles for a specific gauging station (cross-section), and depth and flow velocity were determined using a velocimeter and water samples collected at the surface and at 0.2, 0.4, 0.6, 0.8 and 1 water column depth for each profile. Suspended sediment concentrations were determined by drying at 105 °C until constant weight. Flow rate and suspended sediment delivery rate were determined, respectively, as the products of cross-section area and mean velocity and as flow rate and suspended sediment concentrations. Data on large dams and long-term (1961–2014) rainfall in the Yangtze River Basin and the corresponding analyses are presented in the supporting information. The data used in the paper will be available upon request to lijingbao1951@126.com or ylzhang@niglas.ac.cn.

### Sediment budgets of the two lakes and the ungauged area around TGR

**Figure 1:**
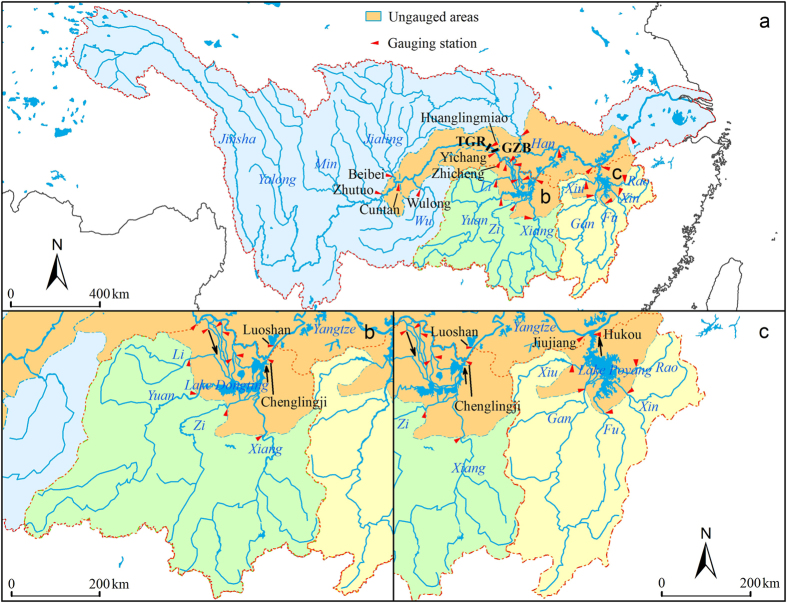
Tributaries, selected gauging stations, ungauged areas and dams in the Yangtze River Basin (**a**), the Lake Dongting Basin (**b**) and the Lake Poyang Basin (**c**), respectively. TGR: Three Gorges Reservoir; GZB: Gezhouba. This map was created using ArcGIS 10.1 software (ESRI Corporation, Redlands, California, USA, https://www.arcgis.com/).

**Figure 2:**
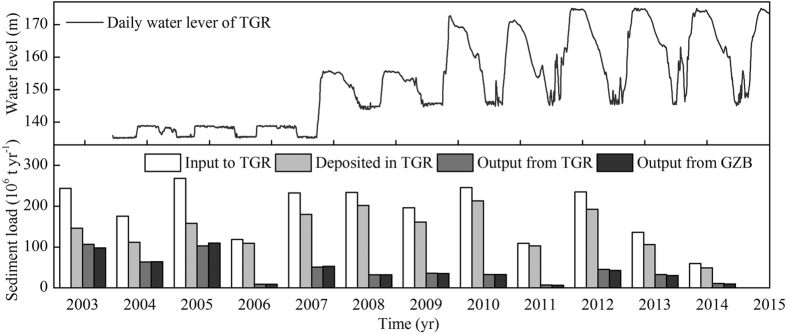
The daily water level of TGR from June 2003 to December 2014 (upper panel; data available at http://www.ctg.com.cn/inc/sqsk.php#1643) and the annual suspended sediment load to and export from TGR and GZB, and the corresponding annual deposition rate (lower panel).

**Figure 3:**
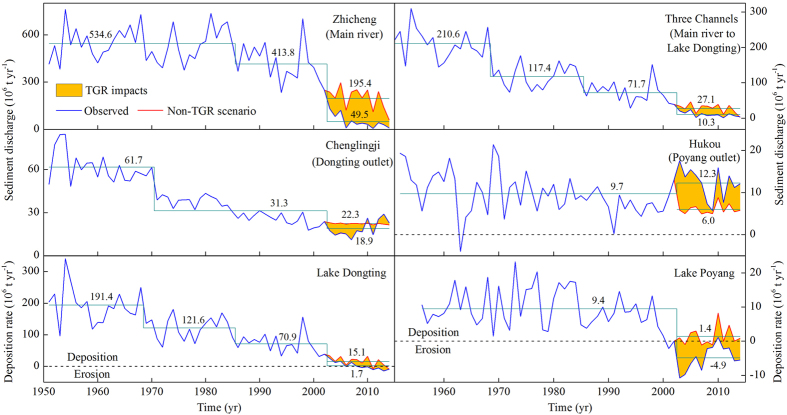
Long-term (1950s–2014) variations of the annual suspended sediment discharge at Zhicheng, the three channels and the Chenglingji and Hukou outlets, and annual sediment deposition/erosion rate in Lake Dongting and Lake Poyang. Also shown is the non-TGR scenario.

**Figure 4:**
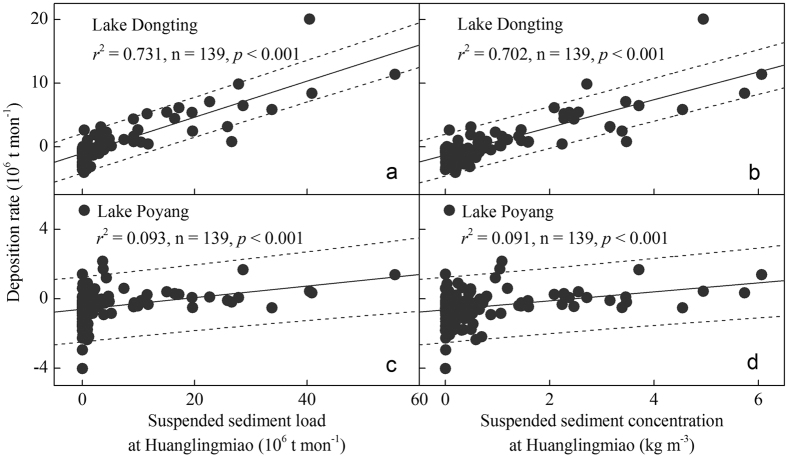
Relationships between monthly (June 2003–December 2014) suspended sediment load, mean suspended concentration in the TGR outflow at the Huanglingmiao outlet and deposition rate of Lake Dongting (**a,b**), Lake Poyang (**c,d**). Dashed lines represent 95% prediction bands.

As the suspended sediment load of Yangtze River at Yichang constitutes >98% of the total sediment flux^19^, we used this as a surrogate for the total sediment flux. In the initial operation period of TGR where its water level was lower than 156 m (June 2003-September 2008; Fig. 2), the upstream limit of TGR was situated downstream of Cuntan and Wulong (Fig. 1) and the downstream limit at Huanglingmiao (<8 km downstream of TGR, Fig. 1), the ungauged area around TGR being 5.32 × 10^4 ^km^2^ (Table S2). We assumed that the sediment yield rates of ungauged small tributaries are similar to those of the neighbouring gauged large rivers^19^. The following equation was used to estimate the suspended sediment load from the ungauged tributaries in the TGR regions (*US*_TGR_) in 2003–2007^19^:


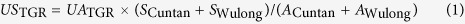


where *UA*_TGR_ represents the ungauged area around TGR and *S*_Cuntan_ and *S*_Wulong_ denote the suspended sediment load from the areas upstream of Cuntan and Wulong, respectively (Fig. 1). *A*_Cuntan_ and *A*_Wulong_ signify the area covered by the gauging stations of Cuntan and Wulong. After October 2008 when the water level of the reservoir became higher than 172 m (Fig. 2), the upstream limit of TGR was extended to the area downstream of Zhutuo, Beibei and Wulong (Fig. 1), and the corresponding ungauged area around TGR was enlarged to 6.84 × 10^4 ^km^2^ (Table S2). The following equation was used to estimate the suspended sediment load from *US*_TGR_ (Fig. 1, panel a) in 2008–2014^19^:


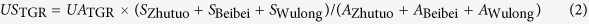


where *S*_Zhutuo_ and *A*_Zhutuo_ represent the suspended sediment load upstream of Zhutuo and the area covered by the gauging station Zhutuo, respectively (Fig. 1). Approximately 19.6% and 22.0% of the Lake Dongting and Lake Poyang watersheds, respectively (Table S2), are not covered by the gauging stations (excluding the water surface area of the lakes). The suspended sediment import to Lake Dongting (*SI*_Dongting_) and Lake Poyang (*SI*_Poyang_) was calculated according to the following equations^19^:


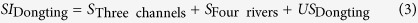



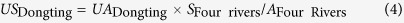



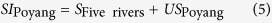



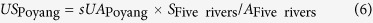


where *S*_Three channels_, *S*_Four rivers_ and *US*_Dongting_ represent the suspended sediment load from the main river to Lake Dongting via the three channels, the suspended sediment load from the four rivers and the ungauged small tributaries in the Lake Dongting Basin (Fig. 1), respectively. *UA*_Dongting_ and *A*_Four rivers_ represent the ungauged areas and the gauged areas in the Lake Dongting Basin (Fig. 1). *S*_Five rivers_ and *US*_Poyang_ are the suspended sediment load from the five rivers and the ungauged small tributaries in the Lake Poyang Basin (Fig. 1). *UA*_Poyang_ and *A*_Five rivers_ denote the ungauged areas and the gauged areas in the Lake Poyang Basin (Fig. 1).

### Sediment budgets in the two lakes for the non-TGR scenario

As in the evaluation by Yang *et al*.^4^,^19^ of the TGR impact on the downstream suspended sediment discharge, we estimated the effect of TGR on the sediment budgets of the two lakes as the difference between the observed and the predicted sediment deposition, assuming a non-TGR scenario in 2003–2014. For the non-TGR scenario, no sediment was presumed retained in the TGR section itself^19^,^25^.

**Figure 5:**
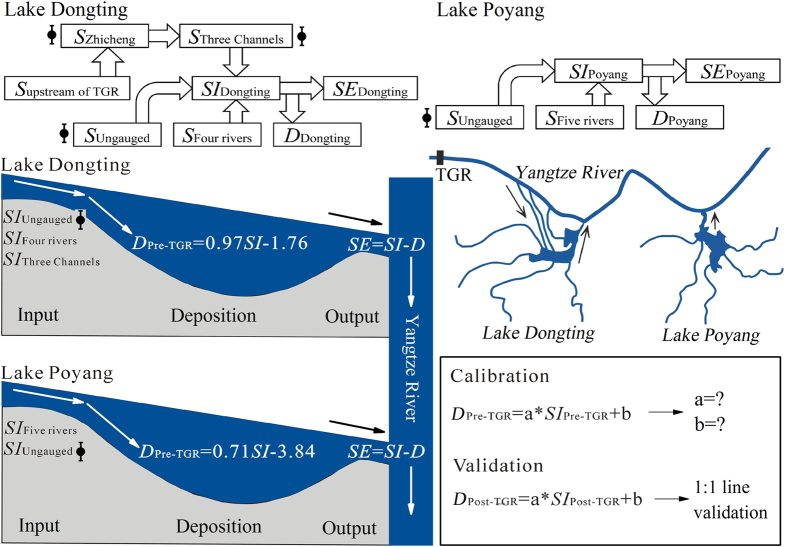
Diagrams showing the river/lake network connections, the sediment budgets of Lake Dongting and Lake Poyang, and how the sediment deposition in the two lakes in the non-TGR scenario period (2003–2014) was calculated including the associated calibration and validation procedures. Error bars denote uncertainties of the sediment budgets of the two lakes for the calculated non-TGR scenario (post-TGR). This diagram was created using CorelDraw Graphic Suite X6 software (Corel Corporation, Ottawa, Canada, http://www.corel.com).

For Lake Dongting, the sediment sources can be categorised into three sub-pools – the three channels, the four rivers and the ungauged small tributaries (Fig. 5). River/lake network connection results indicated that, of the three sources, only the changes in the sediment load from Yangtze River via the three channels after the operation of TGR can be ascribed to anthropogenic factors in the upper reaches of Yangtze River (Fig. 5). We first estimated the suspended sediment load from upstream of Zhicheng (immediately upstream of the three channels, Fig. 1) for the non-TGR scenario during the periods June 2003-September 2008 and October 2008-December 2014 (Fig. 5). To eliminate the impacts of TGR from the non-TGR scenario, the sediment load at Zhicheng during the first period was estimated using the observed sediment load from the area upstream of TGR based on the following Equation (7):


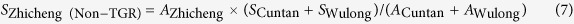


After October 2008, the upstream limit of TGR was extended to the area downstream of Zhutuo, Beibei and Wulong, and another Equation (8) was therefore used to estimate the sediment load at Zhicheng for the non-TGR scenario:


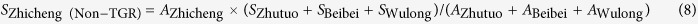


where *S*_Zhicheng_ and *A*_Zhicheng_ represent, respectively, the suspended sediment load from the area upstream of Zhicheng and the area covered by the gauging station Zhicheng (Fig. 1).

A close relationship was recorded between the monthly mean sediment load from Yangtze River to Lake Dongting via the three outlets (*S*_Three channels_) and the main river at Zhicheng (*S*_Zhicheng_) during the pre-TGR period (January 1992-May 2003; Fig. 5; Fig. S9).


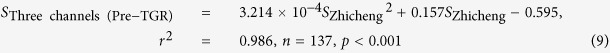


based on Equation (9), we used the estimated *S*_Zhicheng_ for the non-TGR scenario derived by Equations (7) and (8) to estimate *S*_Three channels_ for the non-TGR scenario during 2003–2014^19^,^56^ (Fig. 5). The monthly total sediment input to Lake Dongting (*SI*_Dongting_) for the non-TGR scenario during 2003–2014 was estimated using the following equation:


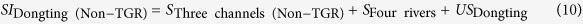


A close relationship was established between the monthly sediment deposition amount of Lake Dongting (*D*_Dongting_) and *SI*_Dongting_ during the pre-TGR period (January 1992-May 2003; Fig. 5; Fig. S9).


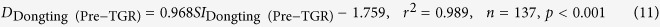


Based on this relationship, we used the estimated *SI*_Dongting_ for the non-TGR scenario derived by Equation (11) to estimate *D*_Dongting_ for the non-TGR scenario during 2003–2014^19^,^56^ (Fig. 5). The sediment export from Lake Dongting to Yangtze River via Chenglingji (*SE*_Dongting_) for the non-TGR scenario during 2003–2014 was calculated using the following equation:


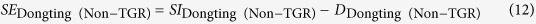


For Lake Poyang, the annual total sediment input to the lake (*SI*_Poyang_) for the non-TGR scenario during 2003–2014 was estimated using the following equation:


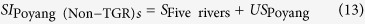


A close relationship was found between the amount of annual sediment deposition of Lake Poyang (*D*_Poyang_) and *SI*_Poyang_ during the pre-TGR period (January 1992-May 2003; Fig. 5; Fig. S9).


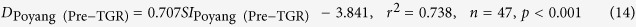


Based on this relationship, we used the observed *SI*_Poyang_ in 2003–2014 derived by Equation (13) to estimate *D*_Poyang_ for the non-TGR scenario during 2003–2014^19^,^56^. The sediment export from Lake Poyang to Yangtze River via Hukou (*SE*_Poyang_) for the non-TGR scenario during 2003–2014 was calculated using the following equation:


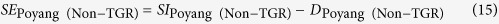


A close relationship was recorded between the daily water level of Hukou (*WL*_Hukou_) and the daily flow rate of Yangtze River at Jiujiang (*Q*_Jiujiang_) during the pre-TGR period (1 January 1999-31 May 2003; Fig. S3).


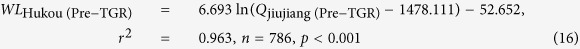


Based on this relationship, we used observed data of daily flow rate of Jiujiang from 1 June 2003-31 December 2014 to estimate the water level of Hukou for the non-TGR scenario in the period. The above empirical relationships (Equations (9), (11), (14) and (16)) for the observed data during the pre-TGR period (1950s–2002) were validated successfully using the observed data in the post-TGR period (2003–2014, see supporting information, Fig. S7).

### Statistical analyses

Linear regressions were performed using MATLAB R2012a and Origin 9.0 software, and *t*-tests were conducted using R-studio software v.0.97.551. Results with *p* < 0.05 were considered significant in the linear regression and *t*-test analyses.

## Additional Information

**How to cite this article**: Zhou, Y. *et al*. Impacts of Three Gorges Reservoir on the sedimentation regimes in the downstream-linked two largest Chinese freshwater lakes. *Sci. Rep.* **6**, 35396; doi: 10.1038/srep35396 (2016).

## Supplementary Material

Supplementary Information
