# Supplementary material for: Impacts of Three Gorges Reservoir on the sedimentation regimes in the downstream-linked two largest Chinese freshwater lakes
Source: Sci Rep. 2016 Oct 17;6:35396. doi: 10.1038/srep35396 (PMC5066318; doi:10.1038/srep35396)
Supplement: Supplementary Information [file srep35396-s1.doc]

## Impacts of Three Gorges Reservoir on the sedimentation regimes in the downstream-linked two largest Chinese freshwater lakes

**(Supplementary information)**

Yongqiang Zhou^1,2,3^, Erik Jeppesen^3,4^, Jingbao Li^5^,*, Yunlin Zhang^1^,*, Xinping Zhang^5^, Xichun Li^6^

^1^Taihu Laboratory for Lake Ecosystem Research, State Key Laboratory of Lake Science and Environment, Nanjing Institute of Geography and Limnology, Chinese Academy of Sciences, Nanjing 210008, China

^2^University of Chinese Academy of Sciences, Beijing 100049, China

^3^Sino-Danish Centre for Education and Research, Beijing 100190, China

^4^Department of Bioscience and Arctic Research Centre, Aarhus University, DK-8600 Silkeborg, Denmark

^5^College of Resources and Environmental Sciences, Hunan Normal University, Changsha 410081, China

^6^Hunan Hydro & Power Design Institute, Changsha 410007, China

^*^Corresponding author: Jingbao Li, College of Resources and Environmental Sciences, Hunan Normal University, Changsha 410081, China, Tel: +86-731-88871451. E-mail: lijingbao1951@126.com

Yunlin Zhang, Nanjing Institute of Geography and Limnology, Chinese Academy of Sciences, 73 East Beijing Road, Nanjing 210008, P. R. China, Tel: +86-25-86882198, Fax: +86-25-57714759.

E-mail: ylzhang@niglas.ac.cn

This manuscript has not been published or accepted elsewhere. We have not submitted it to any other journals. The manuscript is organized according to the format and structure of your journal.

## Dams and rainfall in the Yangtze River Basin

Data on dams in the Yangtze River Basin was obtained from the China hydrological data-sharing service system (http://xxfb.hydroinfo.gov.cn/ssIndex.html?type=3). The water storage capacity of TGR after its full operation (175 m; 393 × 10^8^ m^3^) constituted only 23.3% of the total capacity of all the large dams in the Yangtze River Basin (Fig. S5).

## Long-term rainfall data analyses

Long-term (1961-2014) monthly rainfall data in the Yangtze River Basin with a spatial resolution of 0.5° × 0.5° (longitude × latitude) were obtained from the China meteorological data-sharing service system (http://data.cma.cn/). The monthly rainfall data were first summed into yearly data after which the long-term variations of annual rainfall in the areas upstream of Zhicheng, Lake Dongting Basin and Lake Poyang Basin were calculated using Matrix Laboratory (MATLAB) R2012a software.

Multi-year mean rainfall in the area upstream of Zhicheng decreased from 901 mm in the pre-TGR period (1961-2002) to 873 mm in the post-TGR period (2003-2014, Fig. S6). Similarly, rainfall in the Lake Dongting Basin and Lake Poyang Basin decreased from 1437 mm and 1659 mm, respectively, in the pre-TGR period to 1346 mm and 1576 mm in the post-TGR period (Fig. S6).

| **Table S1** The multi-year mean sediment load (10^6^ t yr^-1^) at Zhicheng, the three channels, the outlets Chenglingji and Hukou, and the deposition/erosion rate (10^6^ t yr^-1^) of Lake Dongting and Lake Poyang during the pre- and post-TGR periods and in the non-TGR scenario (post-TGR), as well as the significance level (*t*-test) of differences between these. | | | | | | | |
| --- | --- | --- | --- | --- | --- | --- | --- |
| Scenarios | Periods | Zhicheng | Three channels | Chenglingji | Hukou | Lake Dongting | Lake Poyang |
| Observed | Pre-TGR | 500.8 ± 124.5 | 134.7 ± 68.2 | 43.0 ± 17.3 | 9.7 ± 4.9 | 130.1 ± 66.6 | 9.4 ± 5.9 |
|  | Post-TGR | 49.5 ± 41.6 | 10.3 ± 7.0 | 18.9 ± 5.6 | 12.3 ± 3.7 | 1.7 ± 12.1 | -4.9 ± 3.6 |
|  | *p* | < 0.001 | < 0.001 | < 0.001 | 0.057 | < 0.001 | < 0.001 |
| Modelled (non-TGR scenario) | Post-TGR | 195.4 ± 71.3 | 27.1 ± 13.0 | 22.3 ± 0.5 | 6.0 ± 1.2 | 15.1 ± 14.8 | 1.4 ± 2.8 |
|  | *p* (vs pre-TGR) | < 0.001 | < 0.001 | < 0.001 | < 0.001 | < 0.001 | < 0.001 |
|  | *p* (vs post-TGR) | < 0.001 | 0.001 | 0.058 | < 0.001 | < 0.05 | < 0.001 |

| **Table S2** Areas (in 10^4^ km^2^) covered by the gauging stations included in the study. | | | | | |
| --- | --- | --- | --- | --- | --- |
| Station | Area | Station | Area | Station | Area |
| Zhutuo | 69.47 | Huangzhuang | 14.21 | Lake Dongting Basin | 26.30 |
| Cuntan | 86.66 | Hankou | 148.80 | Waizhou | 8.09 |
| Beibei | 15.67 | Jiujiang | 152.30 | Lijiadu | 1.58 |
| Wulong | 8.30 | Xiangtan | 8.16 | Meigang | 1.55 |
| Huanglingmiao | 100.28 | Taojiang | 2.67 | Hushan | 0.64 |
| Yichang | 100.55 | Taoyuan | 8.52 | Wanjiabu | 0.35 |
| Zhicheng | 102.40 | Shimen | 1.52 | Lake Poyang Basin | 16.22 |

| 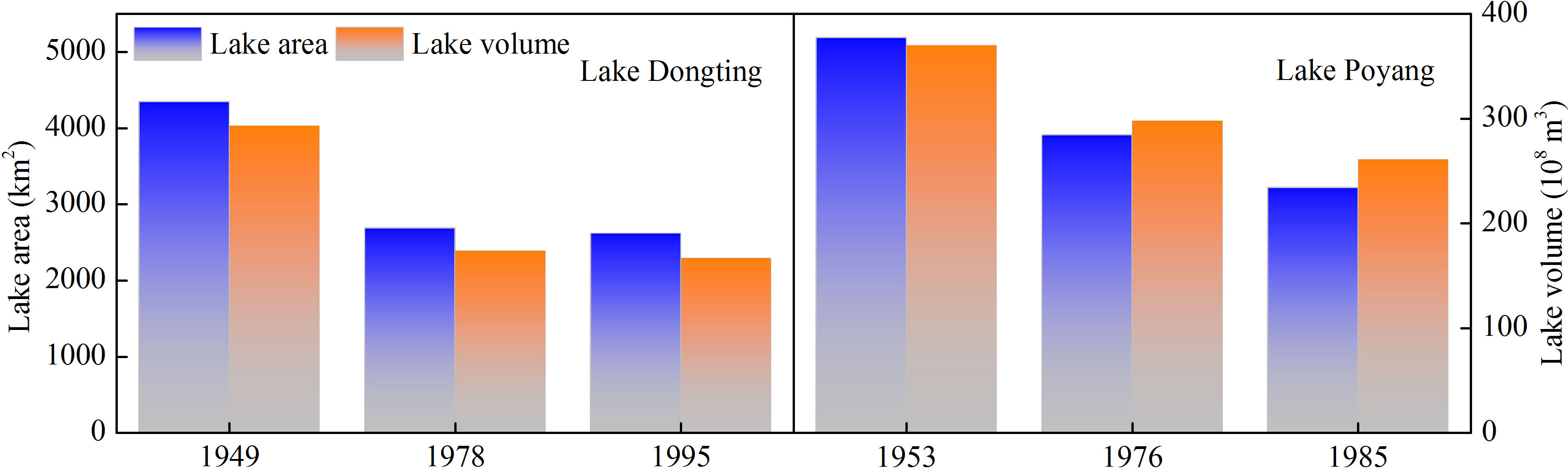 |
| --- |
| **Fig. S1** Variations in lake area and volume of Lake Dongting and Lake Poyang during the past sixty years. |

| 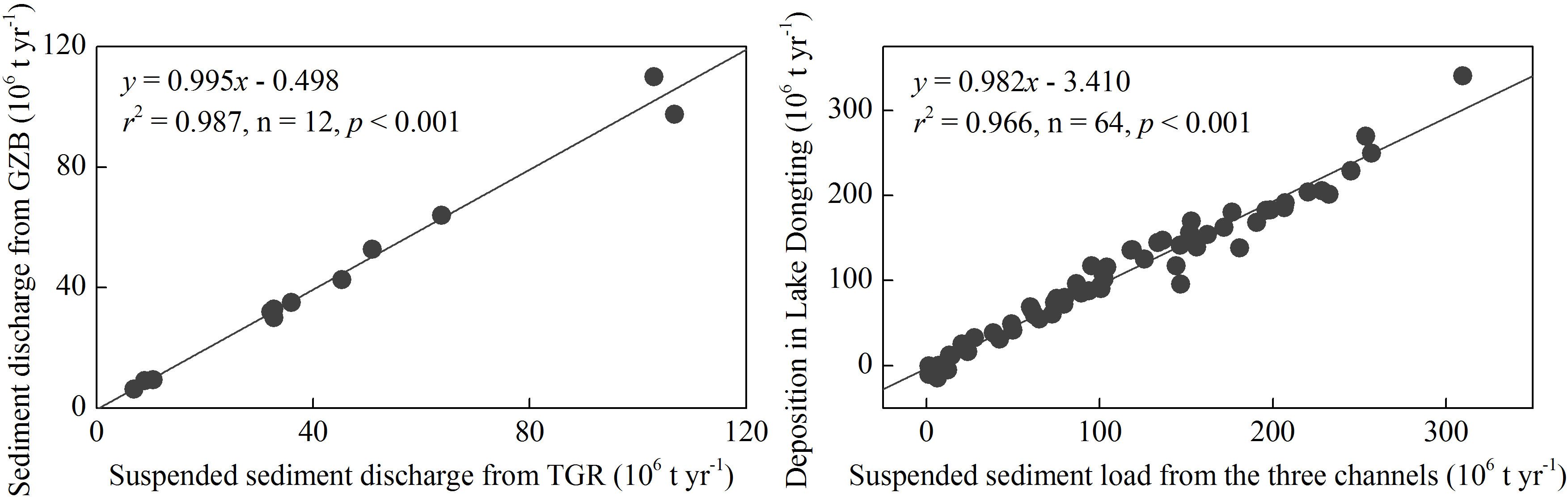 |
| --- |
| **Fig. S2** Relationships between the annual suspended sediment output discharge from TGR and GZB (left panel) and between the annual suspended sediment load from the main river to Lake Dongting via the three channels and the corresponding deposition in Lake Dongting (right panel). |

| 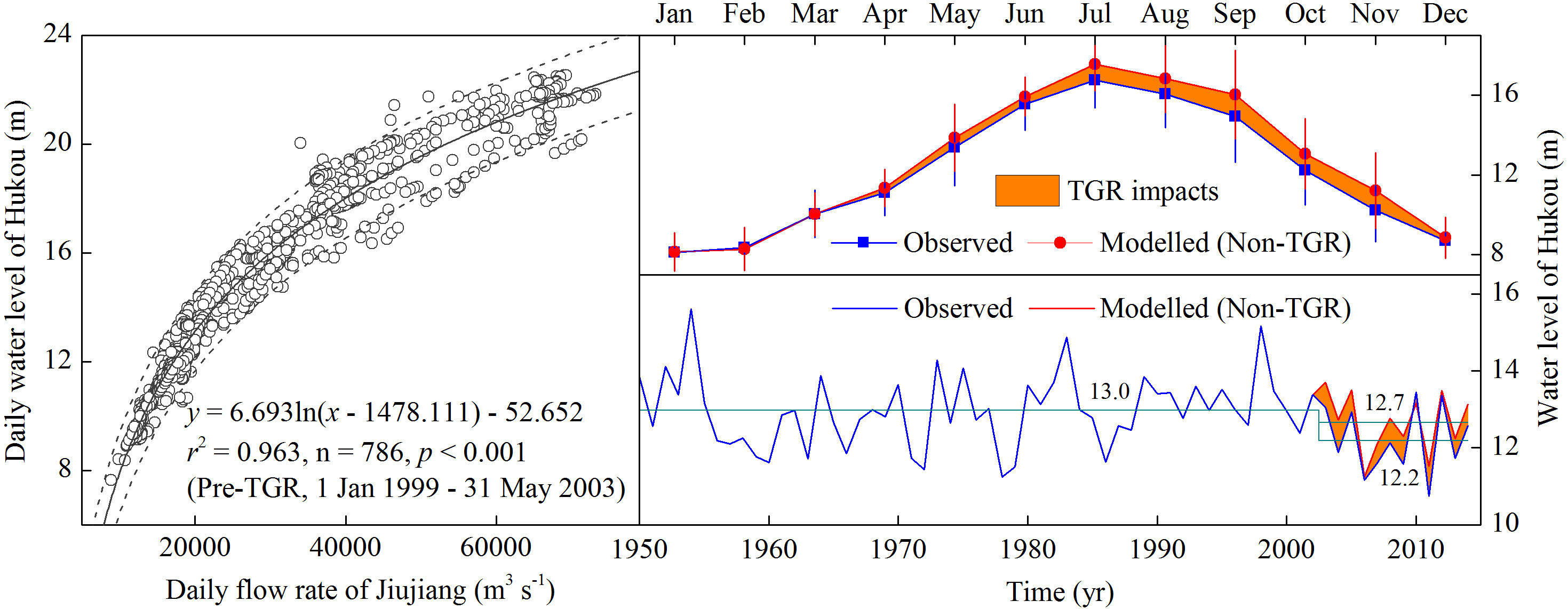 |
| --- |
| **Fig. S3** Relationships between the daily flow rate of Yangtze River at Jiujiang and the daily water level of Hukou in the pre-TGR period (left). Dashed lines denote 95% prediction bands. Observed and modelled (non-TGR case) multi-year mean monthly (upper right) water level of Hukou in the post-TGR period (June 2003 – December 2014). Observed long-term (1950-2014) variations of annual mean water level of Hukou and the corresponding assumed non-TGR case (lower right). |

| 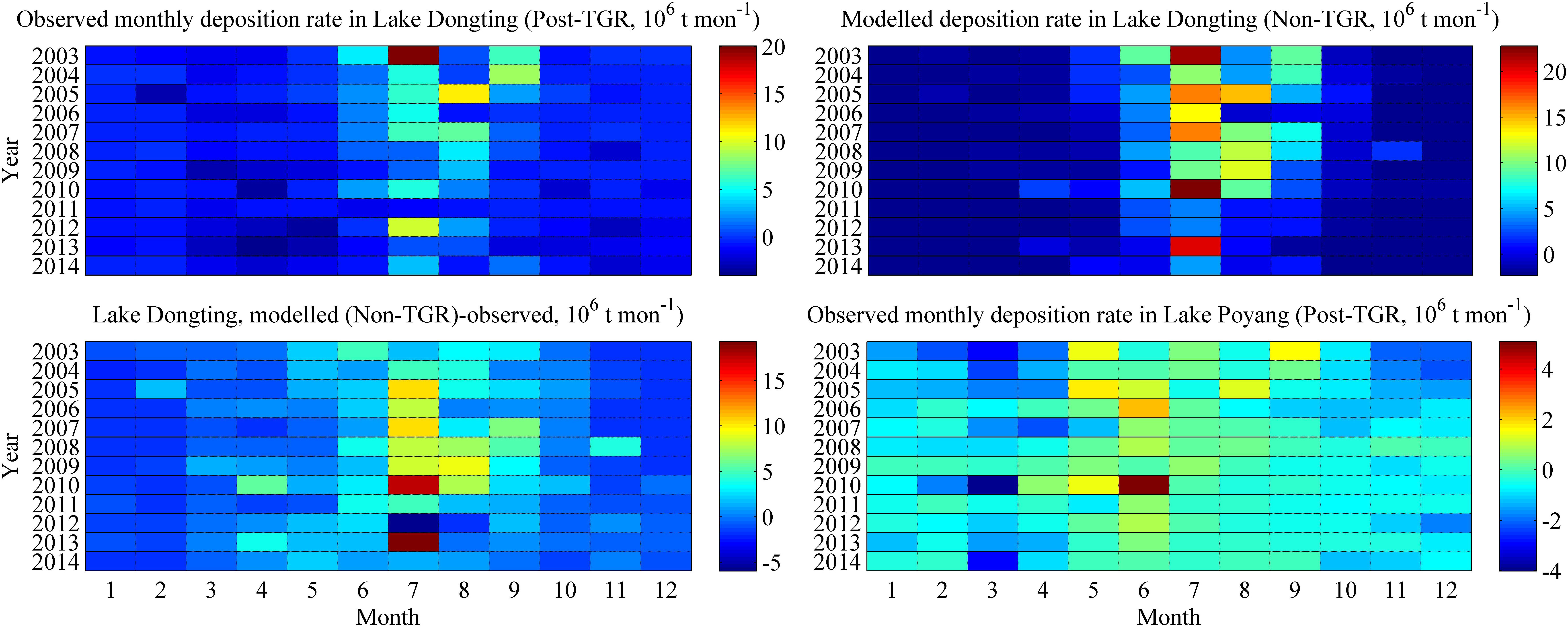 |
| --- |
| **Fig. S4** Observed (upper left) and modelled (non-TGR scenario, upper right) monthly (January 2003 – December 2014) sediment deposition rate in Lake Dongting, the corresponding difference between these (modelled-observed, lower left) and the observed deposition rate in Lake Poyang (lower right). |

| 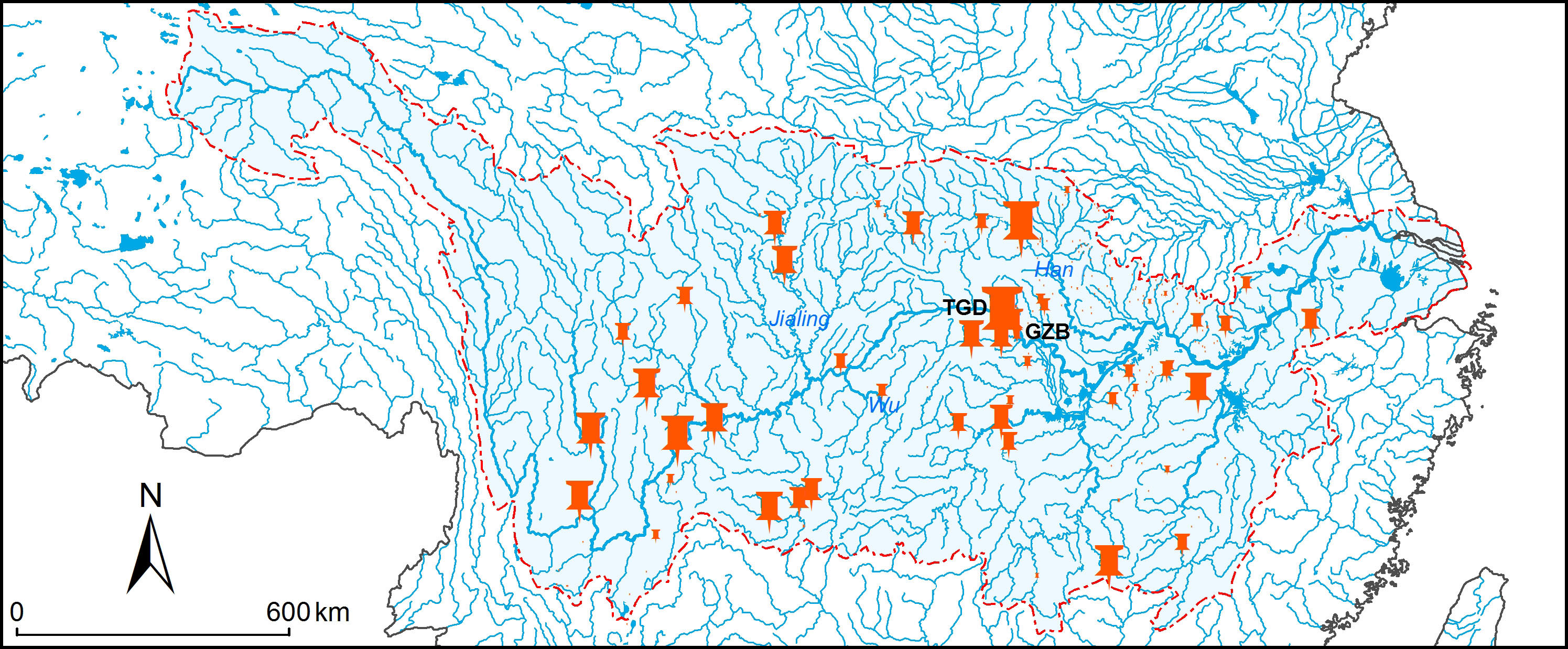 |
| --- |
| **Fig. S5** Distribution of large reservoirs currently in operation in the Yangtze River Basin. The size of the marker reflects the water storage capacity of the individual reservoirs. Data available at http://xxfb.hydroinfo.gov.cn/ssIndex.html?type=3. This map was created using ArcGIS 10.1 software (Esri Corporation, Redlands, California, USA, https://www.arcgis.com/). |

| 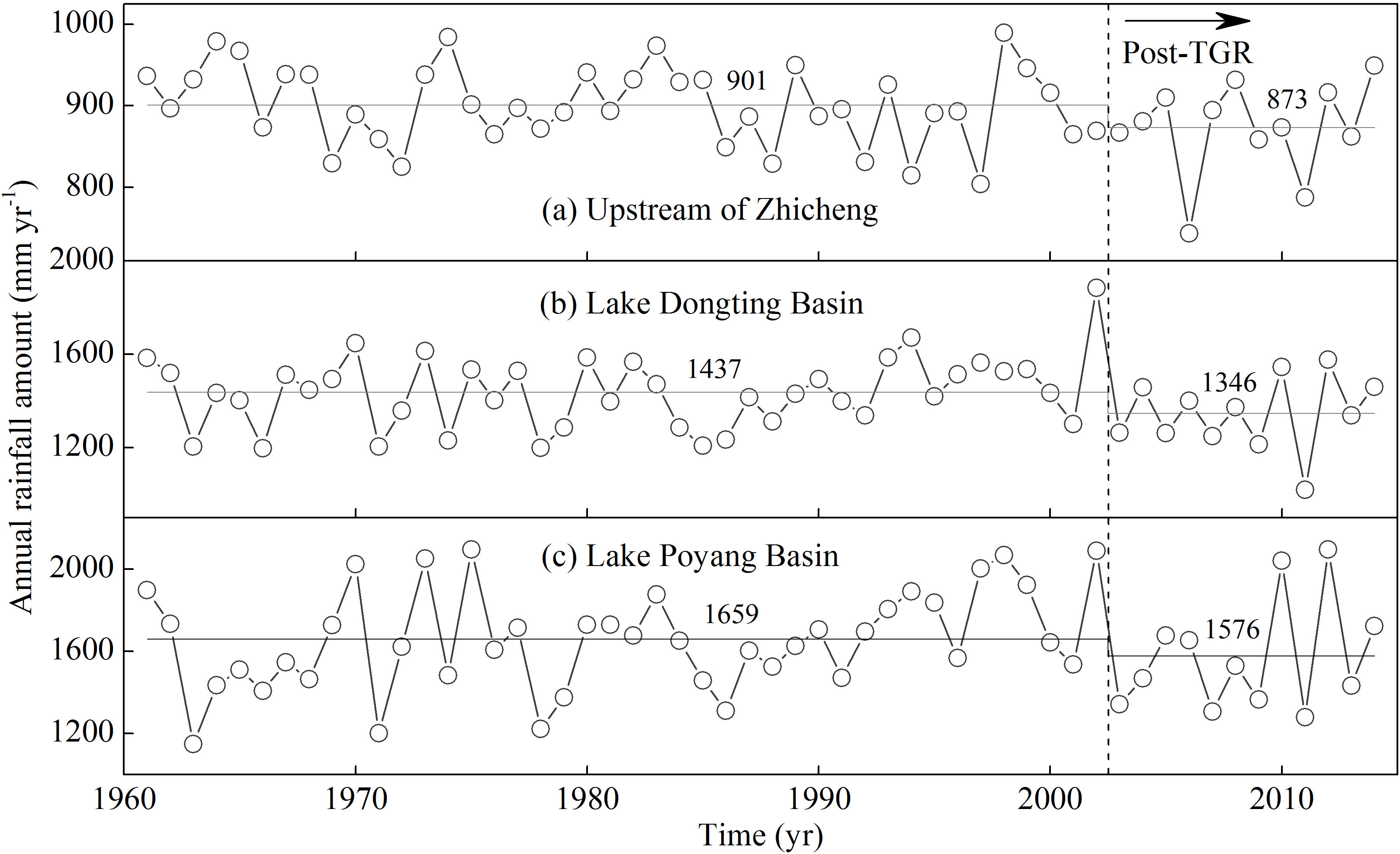 |
| --- |
| **Fig. S6** Annual rainfall in the areas upstream of Zhicheng (a), the Lake Dongting Basin (b) and the Lake Poyang Basin (c) during the pre-TGR (1961-2002) and the post-TGR (2003-2014) period. |

| 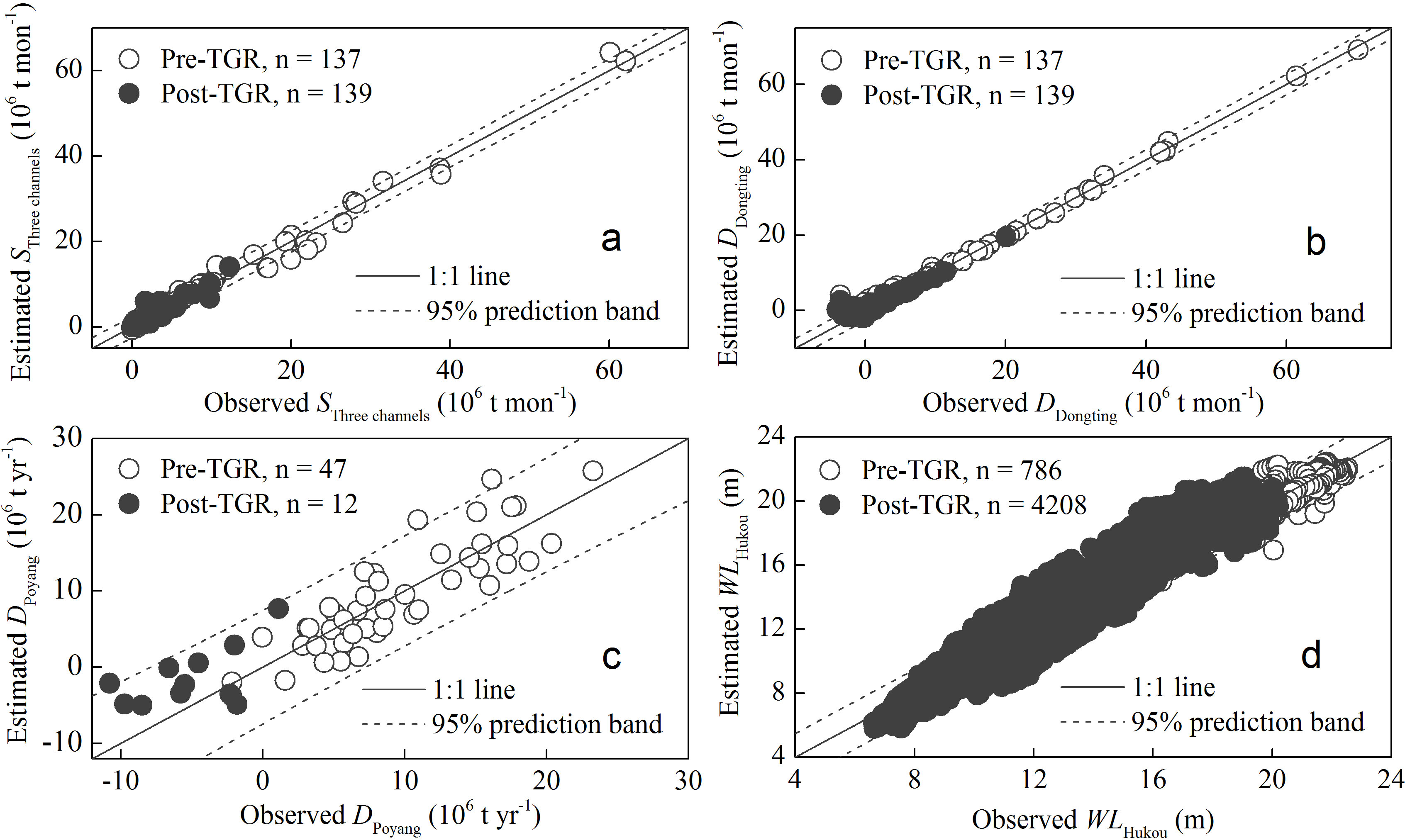 |
| --- |
| **Fig. S7** Validation results for the empirical relationships (equation (9), a; equation (11), b; equation (14), c; and equation (16), d) using the observed data in the post-TGR period. |

| 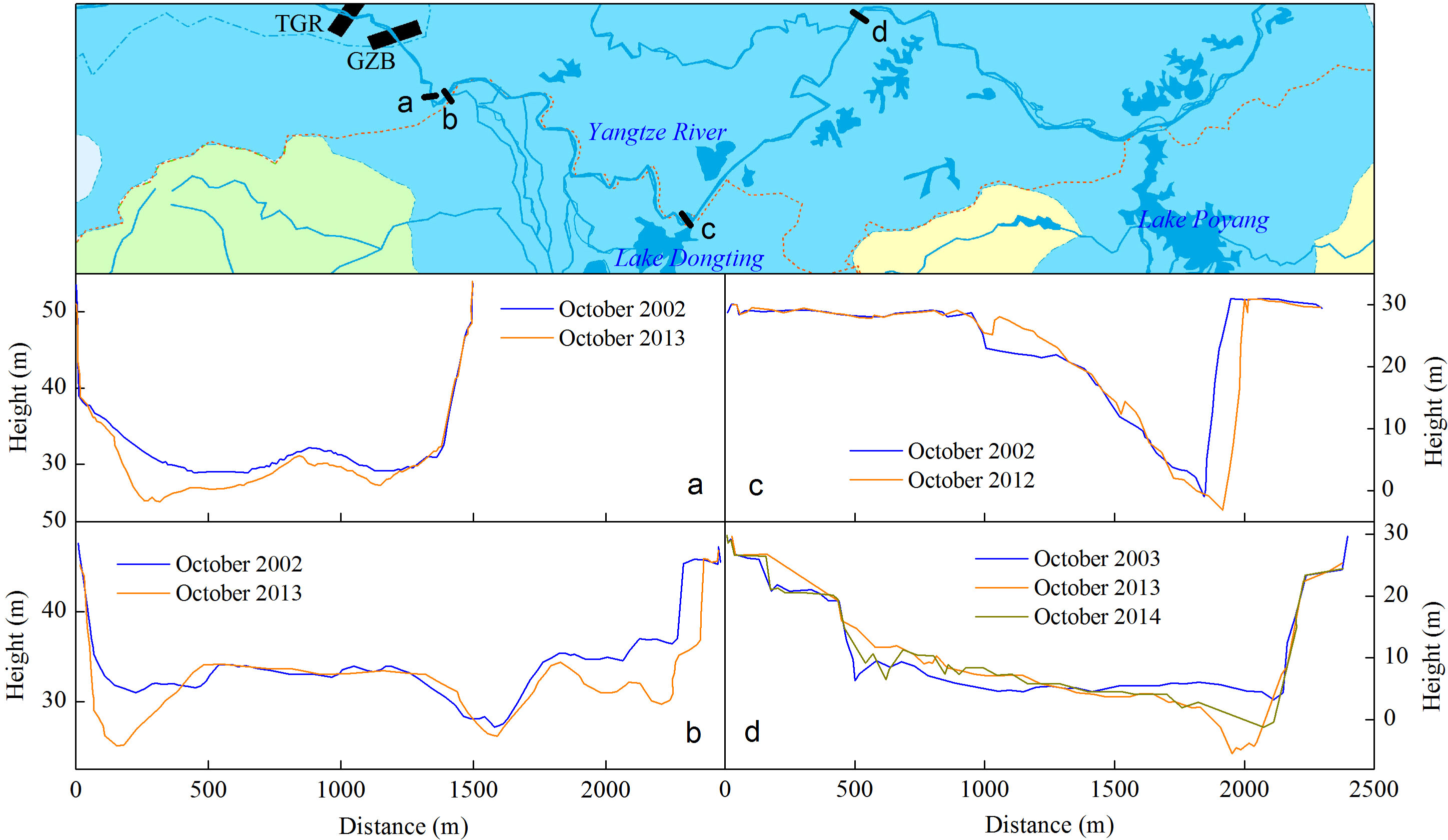 |
| --- |
| **Fig. S8** Cross-channel profiles in the mainstream of Yangtze River based on repeated bathymetric surveys ^1^. The location of profiles a-d is shown on the map in the uppermost panel. The map was created by a combination of ArcGIS 10.1 software (Esri Corporation, Redlands, California, USA, https://www.arcgis.com/) and Origin 8.5 software (OriginLab Corporation, Northampton, Massachusetts, USA, http://www.originlab.com/). |

| 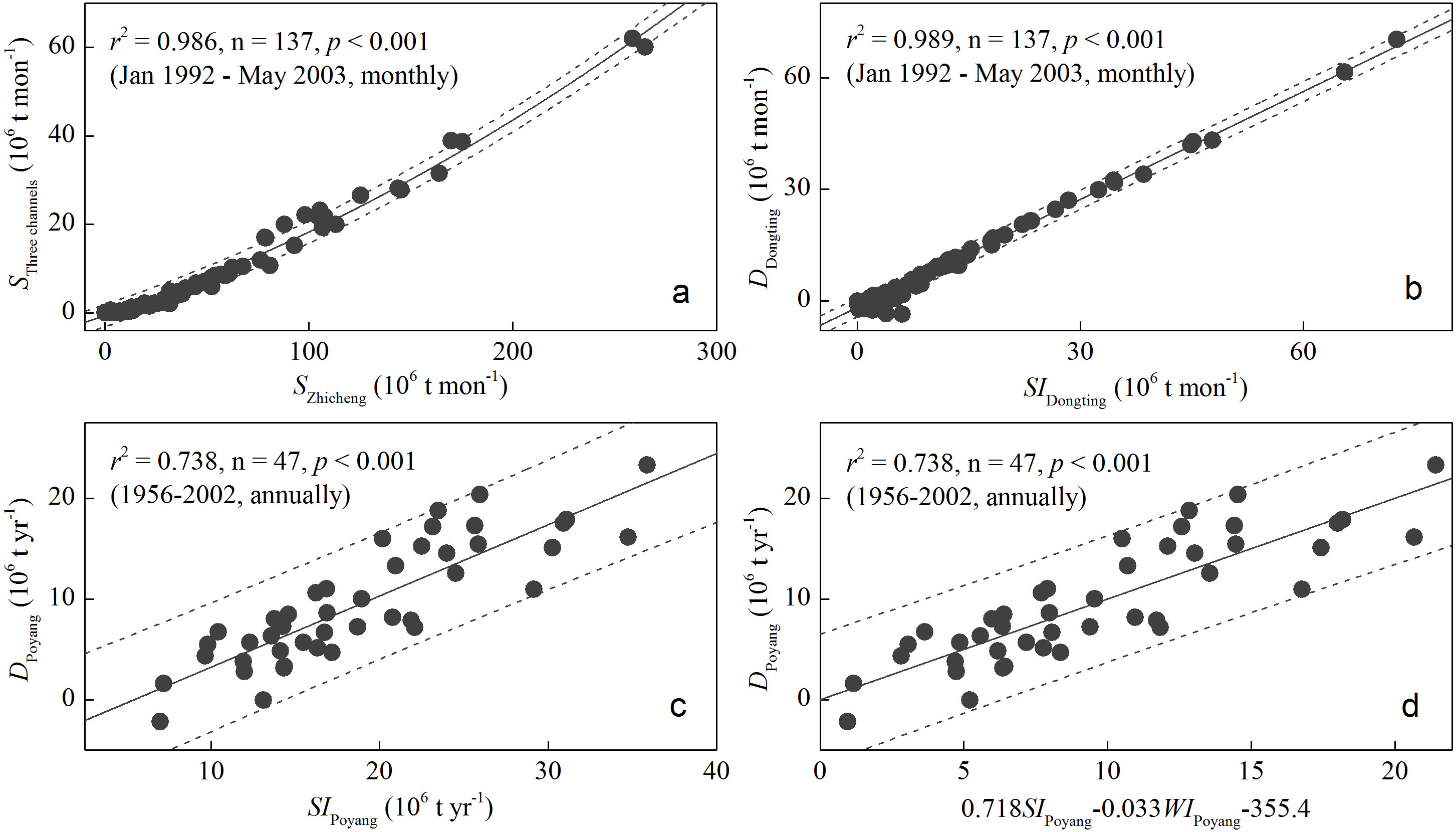 |
| --- |
| **Fig. S9** Relationships between the monthly (January 1992 – May 2003) suspended sediment load of Zhicheng (*S*_Zhicheng_) and the three channels (*S*_Three channels_) (a); total suspended sediment import (*SI*_Dongting_) for the same period and the deposition rate (*D*_Dongting_) of Lake Dongting (b); annual (1956 – 2002) suspended sediment import (*SI*_Poyang_) and the deposition rate (*D*_Poyang_) (c); multiple regression index and the deposition rate (*D*_Poyang_) of Lake Poyang (d). Dashed lines represent 95% prediction bands. |

### References

1 Changjiang (Yangtze) Water Resources Commission (CWRC). Sediment Bulletin of Yangtze River (in Chinese). Available at http://www.cjw.gov.cn/zwzc/bmgb/ (2001-2014).
